# The use of bidirectional rapid reductor in minimally invasive treatment of bicondylar tibial plateau fractures: preliminary radiographic and clinical results

**DOI:** 10.1186/s12891-018-2343-9

**Published:** 2018-11-29

**Authors:** Hengrui Chang, Zhanle Zheng, Yiyang Yu, Jiasheng Shao, Yingze Zhang

**Affiliations:** 1 Department of Orthopaedic Surgery, the Third Hospital of Hebei Medical University, NO.139 Ziqiang Road, Shijiazhuang, Hebei 050051 People’s Republic of China; 2 Key laboratory of biomechanics of Hebei Province, Shijiazhuang, Hebei 050051 People’s Republic of China

**Keywords:** Tibial plateau fractures, Minimally invasive, Traction reduction, Compression bolt, Internal fixation

## Abstract

**Background:**

Minimally invasive treatment of complex tibial plateau fracture represents one of the most challenging problems in orthopedic surgery. We intended to describe the percutaneous surgery involving an originally designed traction device which might facilitate the closed reduction for bicondylar tibial plateau fractures. Further, to assess the clinical outcomes of this minimally invasive technique.

**Methods:**

Between December 2015 and July 2016, Twenty-one patients, mean age 43.71 ± 13.80 years, suffering from a bicondylar tibial plateau fracture (AO/OTA 41-type C) were included. All fractures were firstly reduced by skeletal traction with the aid of bidirectional rapid reductor, and residual depressed fragments were treated with minimally invasive bone tamp reduction. We then evaluated at a minimum follow-up of one year: (1) the rate of complications, (2) the radiographic outcomes (the amount of depression, tibial plateau widening, tibial plateau angle and posterior slope angle) and (3) the clinical outcome (Rasmussen scoring system).

**Results:**

All patients had their fractures healed without secondary displacement. No instrument-related complications occurred during operation. Post-operatively, superficial infection was found in two patients and donor-site morbidity was found in one patient. We observed a < 5 mm step-off in 100% of patients and a < 5 mm plateau widening in 95.5% of patients. Three patients were considered indicative of malalignment with TPA > 90° or PSA > 15°. At last evaluation, the Rasmussen clinical score was excellent in 11 patients (52.3%), good in 9 (42.9%) and fair in 1 (4.8%), and the radiological score was excellent in seven patients (33.3%), good in 14 (66.7%).

**Conclusions:**

The bidirectional rapid reductor facilitates the minimally invasive treatment of bicondylar tibial plateau fracture. The patients exhibited excellent functional recovery. These results should be validated with a larger group of patients and longer period results.

**Trial registration:**

ChiCTR-OPC-16008011.

## Background

Bicondylar tibial plateau fractures (AO/OTA type C) represent 20.4% of all proximal tibial fractures [1, 2]. Because of articular comminution and the frequent occurrence soft-tissue injuries, clinical management still remains challenging for traumatic surgeons [3–5]. Traditional open reduction and internal fixation (ORIF) requires extensive soft tissue dissection, which may lead to numerous soft-tissue complications [6–8]. The Ilizarov technique allows a better choice of closed reduction and fixation which does not necessitate excessive soft-tissue stripping [9]. Today, this technique is being widely used in open and comminuted tibial plateau fractures. However, the external fixation method has its theoretical risks, including pins infection, neurovascular injury, deep venous thrombosis, scar problems, and limitation of joint movement [9, 10]. Besides, frequent management of the circular frame was inconvenient for both patients and doctors.

Recently, several studies have advocated the use of an indirect reduction technique with the aid of knee arthroscopy in tibial plateau fractures. Short- to medium-term clinical outcomes have shown various advantages over ORIF in terms of minimally invasive procedure, accurate reduction monitoring and precise evaluation of intra-articular pathologies [3, 11, 12]. We found this technique to have several limitations. The evidence-based systematic review showed that the majority of included patients had Schatzker type I to III fractures, which suggested that ARIF might have difficulties in managing complex tibial plateau fractures [13]. Lacking of effective traction instruments, varus or valgus malalignment could be hardly reduced through indirect way, especially when knee joint dislocation existed.

Based on the historical experience, the key factor of minimally invasive treatment for such injuries, we considered, was effective and persistent traction which allowed closed restoration of lower-extremity alignment during operation. The primary goal of the present study was to describe a percutaneous surgery involving an originally designed traction device which might facilitate the closed reduction for high-energy tibial plateau fractures. Further, we evaluated the safety of the surgery and analyzed the clinical satisfaction at a minimum follow-up of 1 year after surgery.

## Methods

Between December 2015 and July 2016, 78 patients with tibial plateau fractures were admitted to our emergency department. For this study, skeletally mature patients with an acute, closed, bicondylar tibial plateau fractures (AO/OTA type C) were included in this study. The exclusion criteria were: skeletally immature patients, pathological fractures, concomitant ipsilateral distal femoral fractures, significant pre-existing degenerative joint disease, patients requiring intensive care or with severe systemic illness.

**Fig. 1:**
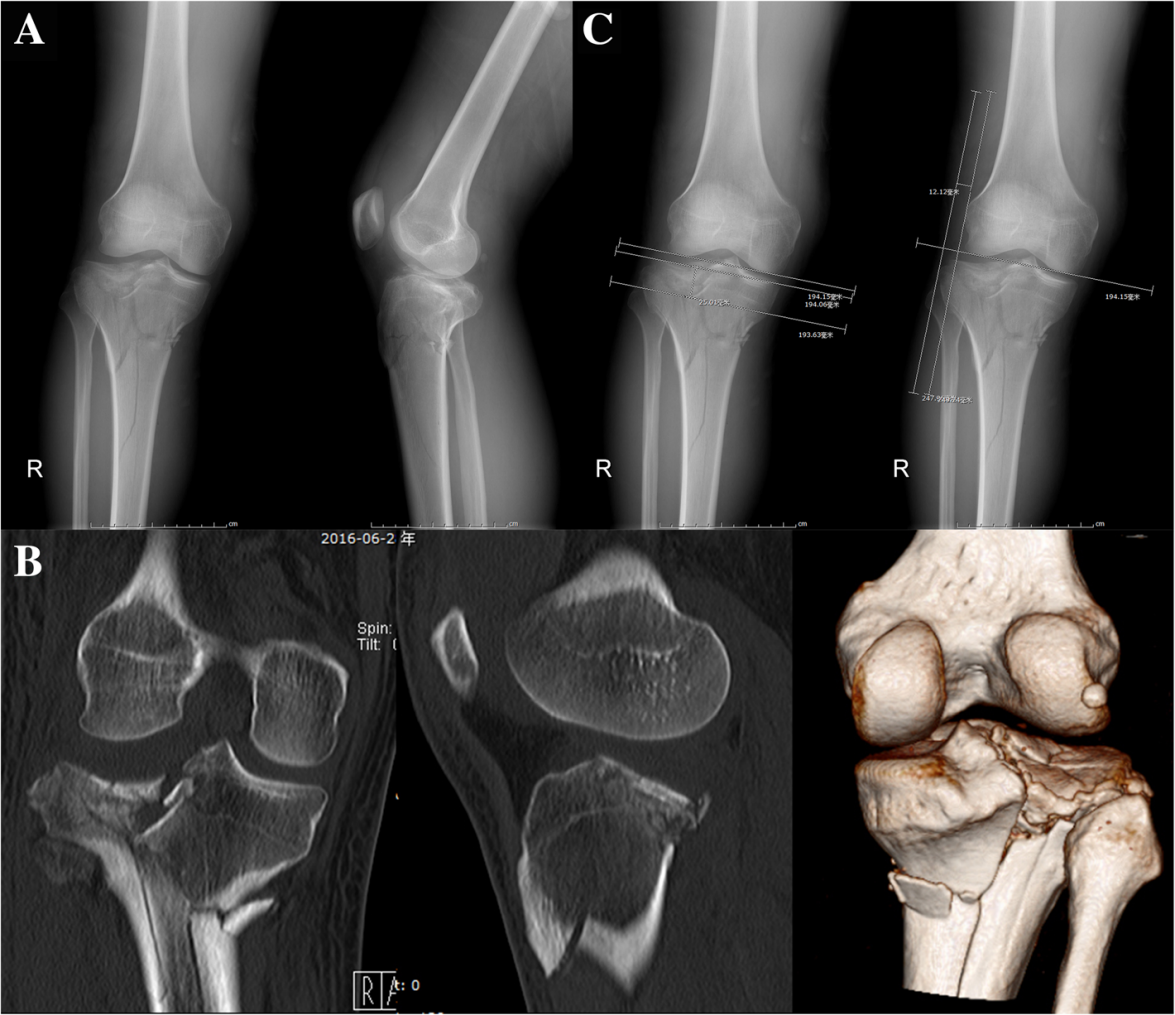
A 34-year-old male sustained a closed complex tibial plateau fracture after automobile collision. Preoperative X-ray images (**a**), preoperative CT images (**b**) and preoperative radiological measurement of articular depression (25.0 mm) and plateau widening(12.12 mm) (**c**)

We identified 21 patients meeting the inclusion/exclusion criteria (Fig. 1a, b). Of the 21 patients included, there were 15 men and 6 women, with 3 left knee injuries and 18 right knee injuries. The average age in this series was 43.7 ± 13.8 years (range 21–67). Most injuries were the results of high-energy trauma including a fall from a height (eight patients), an electric bicycle collision (three patients), an automobile collision (nine patients) and a crushing accident (one patient). 6 patients were associated with other skeletal injuries, and none of which occurred around the injured knee. Basic information, including sex, age, mechanism of injury, and injured side, were gathered from all patients and concluded in Table 1.

**Table 1:** Patient demographics and outcomes, *N* = 21

Case	Age ranges	Sex	Side	AO/OTA classification	Mechanism of injury	Soft-tissue Injuries	Associated skeletal injuries (side)	Follow-up (Months)	Clinical Result (Rasmussen Score)	Radiological Result (Rasmussen Score)
1	35–45	M	R	41-C2	Fall from a height	None	None	20	29	18
2	45–55	M	R	41-C3	Electric bicycle collision	Lateral meniscal tear	None	20	30	14
3	45–55	M	L	41-C3	Fall from a height	Lateral meniscal tear ACL avulsion fracture PCL partial tear	None-	20	30	12
4	55–65	F	R	41-C3	Fall from a height	Lateral meniscal tear	None	19	24	14
5	55–65	F	R	41-C2	Automobile collision	None	Femoral neck fractures (R)	19	28	16
6	30–40	M	R	41-C1	Automobile collision	None	Distal radius fracture (L)	18	30	16
7	60–70	F	R	41-C1	Fall from a height	Medial meniscal tear	None	18	26	18
8	35–45	M	R	41-C1	Automobile collision	None	None	17	27	18
9	45–55	M	L	41-C3	Electric bicycle collision	ACL partial tear	None	17	30	16
10	25–35	M	R	41-C1	Automobile collision	None	Radial fracture (R)	17	28	18
11	45–55	M	R	41-C3	Fall from a height	Lateral meniscal tear Medial meniscal tear	Calcaneal fracture (R)	17	26	14
12	20–30	M	R	41-C2	Automobile collision	None	None	17	24	14
13	35–45	F	R	41-C1	Fall from a height	None	None	17	26	16
14	40–50	M	R	41-C3	Automobile collision	Lateral meniscal tear	None	16	26	14
15	15–25	M	R	41-C3	Automobile collision	Lateral meniscal tear	Ankle fracture (R)	15	30	18
16	30–40	M	L	41-C3	Fall from a height	None	None	15	29	18
17	55–65	M	R	41-C1	Automobile collision	None	None	14	26	16
18	25–35	F	R	41-C2	Automobile collision	None	None	14	30	18
19	30–40	M	R	41-C3	Crushing accident	None	None	13	24	14
20	55–65	F	R	41-C3	Fall from a height	Lateral meniscal tear Medial meniscal tear	Tibial fracture (L)	12	19	14
21	20–30	M	R	41-C2	Electric bicycle collision	None	None	12	26	14

*ACL* anterior Cruciate ligament, *MCL* medial collateral ligament, *M* male, *F* female, *R* right, *L* left

**Table 2:** X-ray measurement of the 21 tibial plateau fractures

	Tibial Plateau Depression (mm)	Tibial Plateau Widening (mm)	Tibial Plateau Angle (°)	Posterior Slope Angle (°)
Preoperation	8.42 ± 8.43	8.06 ± 6.57	NA	NA
Postoperation	1.35 ± 1.59	1.88 ± 1.92	87.86 ± 2.57°	9.76 ± 3.70°
The last follow-up	1.65 ± 1.92	2.05 ± 2.05	87.76 ± 2.98°	10.29 ± 3.41°
p Value	*P*^*1*^ < 0.01; *P*^*2*^ *=* 0.55	*P*^*2*^ < 0.01; *P*^*2*^ *=* 0.79	*P*^*2*^ *=* 0.91	*P*^*2*^ *=* 0.64

*NA* not available

*P*^*1*^ p value of the comparison of pre- and post-operative variables

*P*^*2*^ p value of the comparison of post-operative and the last follow-up variables

During the hospitalization, all patients were treated with transcalcaneus traction for primary alignment maintaining and pain alleviation. Definite treatment was delayed until the soft tissue conditions had improved. Full approval from local Ethics Committee (KE 2016–001-2) was received for this study and all subjects enrolled were confirmed to provide informed consent.

### Surgical protocol

#### Positioning

Patients were in supine position on the radiolucent operating table with a tourniquet placed around the proximal thigh. The tourniquet was inflated to 300 mmHg once the closed traction was complete. Disinfectant preparation covered from proximal 2/3 femoral until to the distal end of lower extremity of injured side. Meanwhile, the anterior superior iliac spine (ASIS) and its surrounding area were also under preparation for the purpose of autogenous bone grafts acquirement. A rolled sheet was placed under the affected knee joint to maintain knee flexion at 30 degrees.

#### Closed reduction in bidirectional rapid reductor

**Fig. 2:**
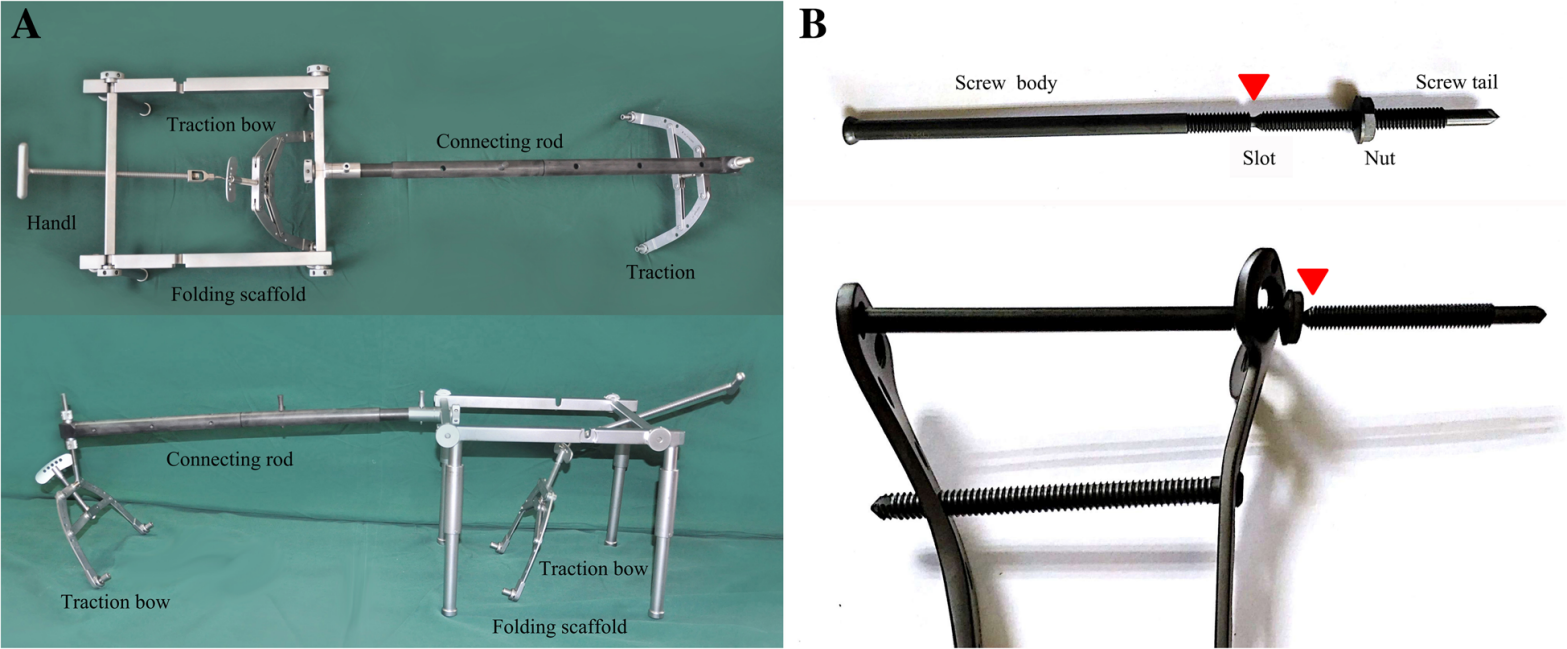
The general view of the bidirectional rapid reductor (**a**) and slot-designed compression bolt (**b**). The compression bolts consist of nuts and slot-designed area (arrow)

**Fig. 3:**
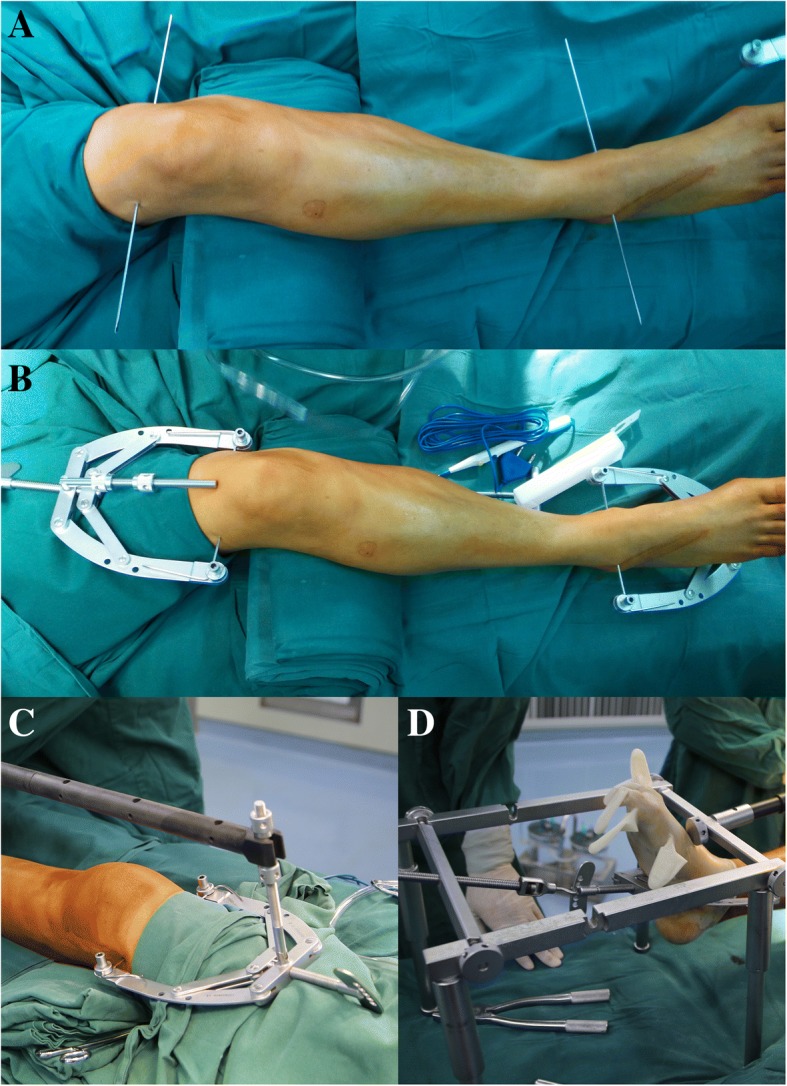
Two 2.5 mm Kirschner wires were firstly inserted through distal tibia and superior femoral condyle (**a**), each Kirschner wire was attached to a traction bow (**b**), The carbon fiber rod connected the proximal traction bow to the scaffold which straddled the lower leg (**c**), the distal traction bow was attached to the rotary handle located on the scaffold (**d**)

The bidirectional rapid reductor is composed of a folding scaffold, connecting rods made of carbon fiber, two traction bows and Kirschner wires (Fig. 2a). All components were able to be packed up into a customized instrument box and were convenient to be sterilized. Two 2.5 mm Kirschner wires were firstly inserted through distal tibia and superior femoral condyle, then, each Kirschner wire was attached to a traction bow (Fig. 3a, b). The carbon fiber rod connected the proximal traction bow to the scaffold which straddled the lower leg and distal traction bow was attached to the rotary handle located on the scaffold (Fig. 3c, d). The scaffold maintained a traction position during the operation. The length of the connection rod could be adjusted to accommodate different patients. Bidirectional rapid reductor was finished assembling all in the sterile field.

**Fig. 4:**
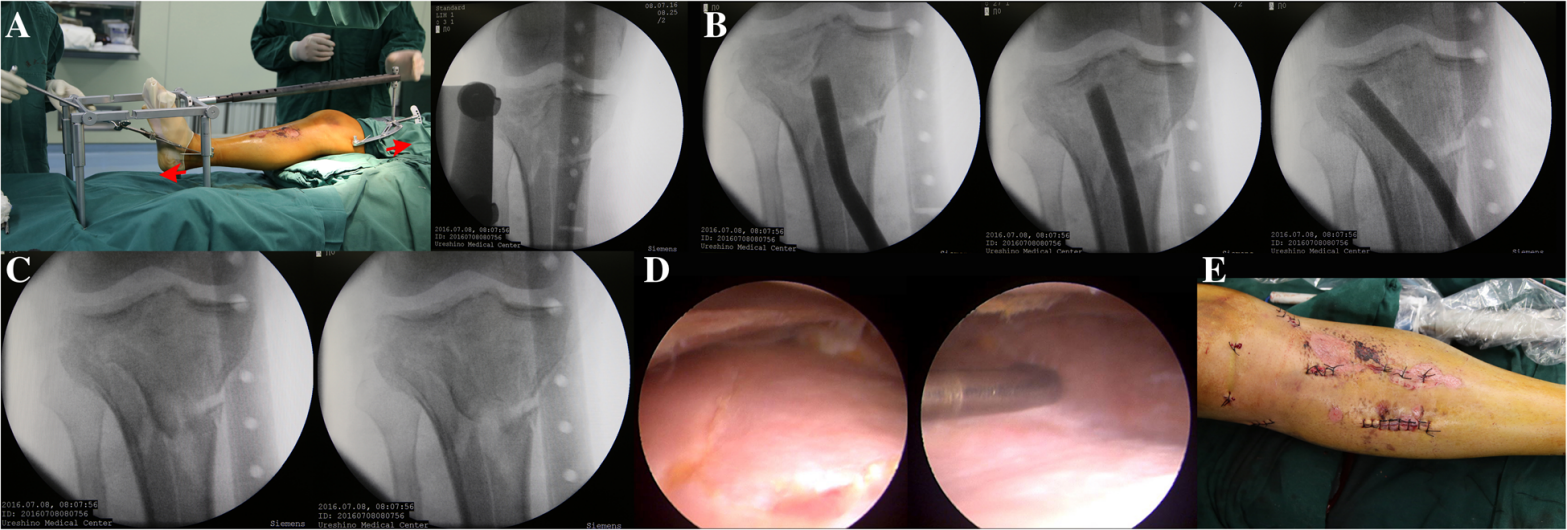
Intraoperative process of the newly proposed treatment. Demonstration of the interaction forces acting on the distal tibia and superior femoral condyle (**a**, arrow), percutaneous reduction of the depressed fragments (**b**), the insertion of autogenous bone graft to support the articular surface (**c**), After reduction and internal fixation under fluoroscopic visualization, arthroscopy revealed a nearly anatomic reduction of lateral articular (**d**), Seven minimal incisions after the operation, four for percutaneous insertion of plates, one for indirect reduction of depressed fragments and two for arthroscopic inspection (**e**)

After confirming that all parts of the reductor were securely joined, a closed-loop traction system was formed by connecting the distal femur, knee joint and distal tibia to the reductor (Fig. 4a). Rotate the handle in a clockwise direction and, as a result, the whole extension force will fall on the distal end of tibia. In the mean time, a counterforce came into being above the knee joint due to the obstruction caused by connecting rod and scaffold. Through ligamentotaxis and skeletal traction, the tibial plateau’s widening, tibial length as well as varus or valgus angulation could be primarily corrected under fluoroscopic guidance. After achieving satisfactory reduction, as revealed by C-arm examination both on anteroposterior (AP) and lateral views, the reductor could sustain the lower extremity in a reduction position. The whole traction process was achieved on the operating table and no special positioning of patient was needed, which largely facilitated the placement of fluoroscopy monitors.

#### Elevation of depressed articular fragments

For patients with articular depression, care must be taken to locate the orientation of depressed fragments based on preoperative x-rays and CT scan. A 2.5 mm K-wire was chosen as a guide for creation of a tunnel. The ideal entry point was 2 cm below the centre of tibia tubercle, slightly medial or lateral to the long axis of tibia which depends on the location of depressed fragments. Mostly, the medial approach was used for the management of lateral plateau depression. A longitudinal 3 cm skin incision was made on the medial aspect of the tibia around the K-wire. Then, a cortex portal was made, and the depressed fragments was elevated with a customized bone tamp via the inferior transosseous tunnel created by step drills. The whole reduction process is performed with gentle tapping of the bone tamp under the inspection of AP and lateral views of fluoroscopy (Fig. 4b). Percutaneous reduction was optimized until intraoperative fluoroscopy suggested satisfactory articular reconstruction without a remaining articular depression. Subsequently, the Autogenous bone graft harvested from the iliac crest was then inserted into the bone tunnel to support the subchondral bone and articular surface (Fig. 4c).

#### Internal fixation

Once the reduction was obtained, minimally invasive percutaneous plate osteosynthesis (MIPPO) was performed using pre-contoured locking compression plate (LCP) designed for proximal tibia (WEGO, Wei Hai city, PR China). In all cases, dual plate fixation was used medially and laterally, and no additional plate was needed. Subsequently, one or two compression bolts designed with special slot (WEGO, Wei Hai city, PR China) would be inserted through the most proximal holes of two plates (Fig. 2b), which permitted further restoration of the tibial plateaus’ width. Once the nut passed through the slot, the bolt’s tail could be easily broken off at this constricted area with appropriate size left. Care was taken to ensure the alignment was not changed on the AP views when tightening the left screws.

#### Arthroscopic evaluation

Following the optimal fixation, the bidirectional reductor was disassembled and arthroscopic examination was then performed using gravity inflow and without incision sutured. The heamarthrosis was drained and any osteochondral fragments were removed. Previous reconstruction of the articular surface and soft tissue injury was then evaluated (Fig. 4d, e).

### Postoperative management

**Fig. 5:**
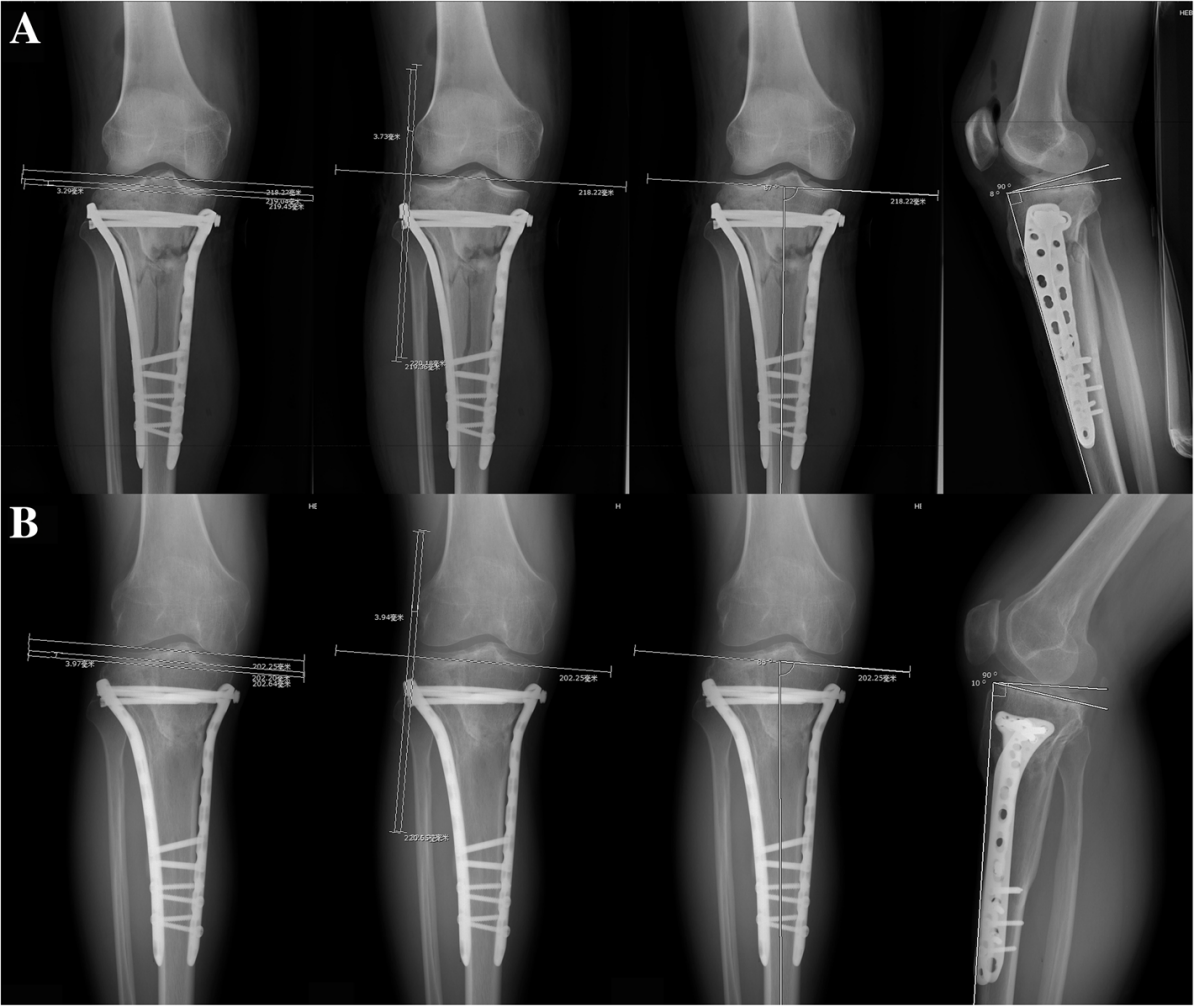
The postoperative measurements of step-off (3.29 mm), plateau widening (3.73 mm), TPA (87°) and PSA (8°) (**a**). At the last follow-up, the radiological measurements the step-off (3.97 mm), plateau widening (3.94 mm), TPA (85°) and PSA (10°) (**b**)

Immediate non-weight bearing joint motion was encouraged as the pain subsided. Based on general condition and post-operative X-ray examinations, progress weight-bearing usually started with crutches at 8 weeks. Peri- and post-operative complications such as wound infection, thrombosis, compartment syndrome, donor site pain and joint stiffness were recorded. Independent knee assessments were carried out post-operatively using the Rasmussen’s clinical and radiologic scores after a mean duration of follow up of 16.5 months (range, 12 to 20 months). The amount of fragment depression, plateau widening, TPA (tibial plateau angle) and PSA (posterior slope angle) were measured on the preoperative, postoperative radiographs and at the time of the latest follow-up on the basis of X-ray measurement (Fig. 5a, b).

### Statistical methods

Statistical analysis was performed using IBM SPSS Statistics 21 (SPSS Inc., Chicago, IL). Data are presented as mean values ± SD for continuous variables. The normality of the distribution of the parameters was tested using the Kolmogoroff-Smirnoff test. Continuous variables with non-normal distribution were analyzed using the Mann-Whitney U test (preoperative/postoperative/follow-up radiological outcomes). All tests were 2-sided and a *P* value of < 0.05 was considered significant.

## Results

Six patients suffered from OTA/AO 41-C1, 5 from 41-C2 and 10 from 41-C3 fractures. With the help of rapid reductor, all 21 cases of bicondylar tibial plateau fractures were reduced in a closed fashion, and none converted to open reduction. The average operation time was 100 min (excluding the time of arthroscopic examination, range 50 to 150 min, SD 29.4). The average blood loss was 135 mL (range 80 to 300 min, SD 58.1). Through arthroscopy, an intra-articular diffusion of cancellous bone debris was observed in three patients. The fragments were all removed using nucleus pulposus clamps and were not registered as a complication. Two cases with high energy fractures developed superficial infections, which delayed the wound healing, and were managed with a course of oral antibiotics for a week. There were no episodes of wound dehiscence, deep venous thrombosis or compartment syndrome at the time of hospital discharge. No patient subsequently developed deep infection, osteomyelitis or joint stiffness at follow-ups. One patients who received autogenous bone grafts sustained donor-site morbidities. However, this patient did not require further intervention for pain relieve because of their well tolerance of the pain. No other adverse outcomes were occurred during follow-up.

In 16 of 21 fractures, autogeneous bone grafts were implanted to provide mechanical support for the reduced fragments. Associated soft tissue injuries were common, occurring in 9 (42.9%) patients. Arthroscopy revealed tears of the lateral meniscus alone (*n* = 5), the medial meniscus alone (*n* = 1), or a combination of both meniscus (*n* = 2). There were three lateral meniscal repairs and five partial meniscectomy performed. One partial meniscal tears required conservative treatment. There was one partial ACL (anterior cruciate ligament) ruptures, one partial PCL (posterior cruciate ligament) ruptures, and one avulsion fracture of PCL. Considering the displacement was minimal, the eminence avulsion and other incomplete ligament injuries were all treated with the immediate cast-brace fixation. Using the distal tip of probe (length = 3 mm) as reference, arthroscopy showed a persistent > 3 mm fracture depression in three patients and a > 3 mm fracture gap in one patient. However, in these cases, all the displacement was less than 6 mm (twice the length of probe tip) and without further intervention.

All 21 patients were followed up at a mean of 16.5 ± 2.5 months (range, 12 to 20 months) postoperatively. At last follow-up, all patients had healed their fractures with no loss of reduction. The mean depressed step-off of the lateral joint decreased from 8.42 ± 8.43 mm (0–26.60 mm) pre-operatively to 1.35 ± 1.59 mm (0–4.49 mm) post-operatively (*p* < 0.01). The mean lateral plateau widening decreased from 8.06 ± 6.57 mm (0–29.35 mm) pre-operatively to 1.88 ± 1.92 mm (0–5.4 mm) post-operatively (*p* < 0.01). The mean TPA and PSA were 87.86 ± 2.57° and 9.76 ± 3.70°, respectively. We observed a ≤ 2 mm step-off in 68.2% (15/22) of patients and a > 2–5 mm step-off in 100% of patients. We also observed a < 5 mm plateau widening in 95.5% of patients with only one patient having a widening > 5 mm (5.4 mm). 3 cases (14.3%) of malalignment of proximal tibia were identified in this group, two of which involved in coronal plane (TPA: 92°; 94°) and one in sagittal plane (PSA: 16°). These radiological results were stable until the last follow up after surgery. We found a mean lateral step-off of 1.65 ± 1.92 mm (*p* = 0.55), plateau widening of 2.05 ± 2.05 mm (*p* = 0.79), TPA of 87.76 ± 2.98° (*p* = 0.91) and PSA of 10.29 ± 3.41° (*p* = 0.64) (Table 2). Finally, the mean radiographic Rasmussen score was 15.71 ± 1.93 (12–18), with seven patients (33.3%) rated as excellent and 14 patients (66.7%) rated as good.

**Fig. 6:**
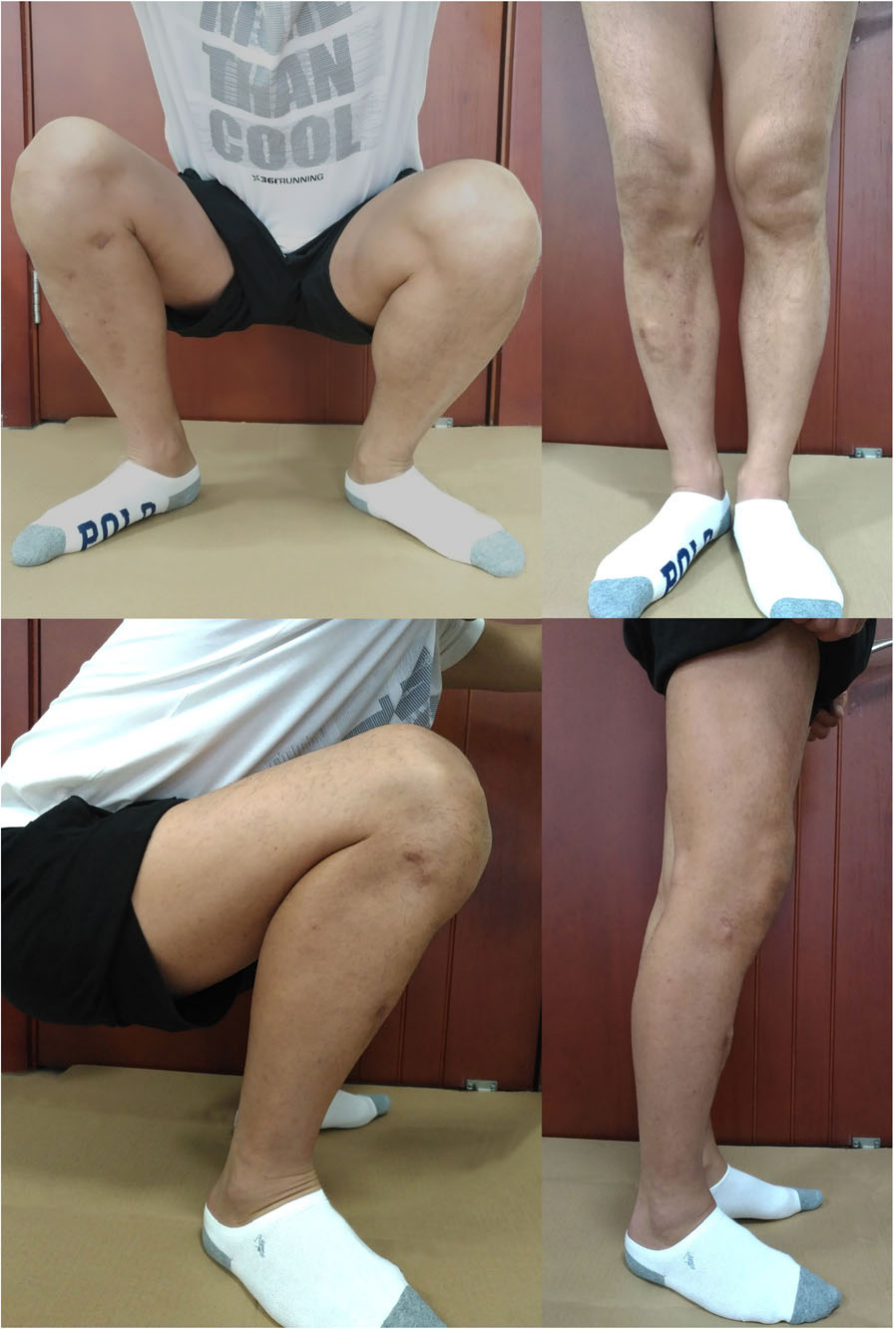
Pictures of the knee function of the case demonstrated in Figs. 1, 4 and 5 at the final review 13 months after the accident

The mean Rasmussen clinical score was 27.05 ± 2.84 (19–30). Eleven patients (52.3%) were classified as excellent, nine (42.9%) were classified as grade good, one (4.8%) were classified as fair. The patient who was rated as fair had residual depression of 4.5 mm of the lateral tibial plateau, with mild valgus deformity (TPA: 94°). This patient complained of intense, constant pain around the lateral compartment after activity. The range of motion at last follow-up was mean flexion 129.29 ± 8.70° (115–140°) and mean extension 1.95 ± 3.47 (0–11°) (Fig. 6).

## Discussion

In the present study, we treated 21 bicondylar tibial plateau fractures with bidirectional traction device and slot-designed compression bolts in a minimally invasive manner. The rapid reductor could not only efficiently reduce the fracture but also maintained it in an anatomical position. Anatomic or nearly anatomic reduction of the articular surface, tibial plateau width, TPA and PSA was achieved in most cases. This minimally invasive procedure offered advantages of low soft-tissue related complications and less operation time. The technique itself was safe without any instrument-related adverse case. All fractures healed well and satisfactory functional recovery was identified in the majority of the patients.

Given the soft tissue damage and comminuted fracture pattern of OTA/AO type C tibial plateau fractures, surgical treatment has been associated with a high complication rate, specifically postoperative deep wound infection [5, 14]. Previous studies have shown that long-term outcome depended more on the stability of the knee and that less extensive operations might lead to satisfactory results [15, 16]. Unfortunately, alignment and stability were not easily controlled in the treatment of complex fractures of the tibial plateau, particularly when minimally invasive surgery was required. Some surgeons preferred to use a fracture table or other skin traction device to treat tibial plateau fractures in a closed way. However, its function on managing articular depression was limited [17, 18]. Ilizarov fixation allowed distraction, which aided reduction of the peri-articular fragments when applied along the lower limb. Clinical studies showed good long-term functional results in high-energy tibial plateau fractures [9, 19]. However, frequent pin-site infections and potential risk of later limitation of movement have been notified even in experienced hands [9, 20, 21]. The overall incidence of pin-site infection was reported to be 23.9 to 53.3% [9, 10, 22, 23]. Besides, postoperative adjustment of the external fixation required additional workload both for patients and doctors.

Different from skin traction, the bidirectional traction device could generate enormous longitudinal force applied on the distal end of tibia. Meanwhile, an equivalent counterforce came into being because of the obstruction by the connection rod. This is what so called “bidrectional traction” [24]. The closed-loop traction system could able to maintain the lower limb in a traction condition from the beginning to the end of surgery. Through skeletal traction, ligamentotaxis and soft-tissue (capsule, ligaments and muscles) compression, the widening displacement and malalignment could be partially reduced in a closed way. Compared with manual traction or distractor, the traction force generated by the rapid reductor was much more effective, sustained and symmetrical. Instead of arthrotomy, traction-based reduction was easier to perform which resulted in shorter operative time and less blood loss. In the present study, the average operative time and blood loss were 100 min and 135 ml, respectively. By the method, all 21 AO/OTA type C fractures gained satisfactory reduction initially.

We elevated any depressed segments with a customized bone tamp under the guidance of fluoroscopy. The methods mentioned above was similar to the “Medial Approach” technique which was firstly adopted by Levy et al. [25]. Benefit from the compression of tensive soft-tissue, the tibial plateau remained stable and was unlikely to be wider when depressed fragment was elevated to its anatomic position. With the application of intraoperative arthroscopy, we were able to demonstrate that fracture reduction was successful in 82% patients leaving a fracture depression or gap < 3 mm, Finally, our radiological outcomes are promising as we obtained a ≤ 2 mm step-off in 68.2% (15/22) of patients and a > 2–5 mm step-off in 100% of patients.

As another study putted, we also acknowledged that the depressed fragments could not always be anatomically restored especially when the articular was severely damaged [3]. This could be handled with meticulous pre-operative preparation and careful manipulation maybe with frequent X-ray exposure at first. Therefore, surgeons should take notice of the necessity of radiation protection. Krause et al. recommended arthroscopy should be indicated in comminuted tibial plateau fractures [4]. In this study, the articular reconstruction visualized by arthroscopy was consistent with fluoroscopic results. Although a residual step-off or fracture gap was existed in four cases, we decided no further management at the sacrifice of further soft-tissue stripping and longer operation time.

Slot-designed compression bolt was another key instrument which had dual functions of both reduction and fixation. We once applied the same technique in managing calcaneal fractures and received satisfactory outcomes [26–28]. In fact, the laterally displaced fragments were difficult to reduce simply by traction. The compression bolt which played as the bridge of lateral and medial plate could provide efficient compression force to further restore the width of tibial plateau. When the nut was tightened, the compression force did not act on the bone facet directly, but on the dual plates and then transmitted to tibial plateau. The mechanical transmission avoided stress concentration and hardware-related complications including osteoclasia and osteolysis. The unique slotted design facilitated the removal of the distal part of screw without a residual tail left, which decreased the risk of hardware irritation beneath skin. Meanwhile, the compression force generated by the bolts lasted for a long time until the fracture was healed which was crucial for preventing further displacement when early mobilization and weight bearing carried on. The radiological results in the present study were stable until the last follow up without any case of loss of reduction or alignment.

Using the Rasmussen criteria for radiographic assessment, excellent to good outcome was achieved in all our cases, which is comparable to the similar series treated with Ilizarov technique (90 to 100%) [22, 29]. When applying the Rasmussen clinical rating system, the average score in our study was 27.1 and excellent to good outcome was achieved in 95% patients. According to the same rating system, Barbary et al. reported a similar functional result of 93% good to excellent satisfaction after the Ilizarov treatment [29]. In a series of 59 complex plateau fractures, Catagni et al. reported a result of 96.6% good to excellent satisfaction after Ilizarov fixation [10]. Based on these evidences, we suggested that the percutaneous treatment, according to our technique of rapid reductor, could achieve similar clinical results compared with Ilizarov technique. Complications in the current series were minimal because of the obviation of traditional arthrotomy and easier manipulation. No case of joint stiffness, pin tract infection, deep infection or other severe event happened.

Some limitations to this study should be acknowledged. First, this study reports a small series of patients with bicondylar tibial plateau fractures. Besides, there was no control group with which to compare our results. However, the goal of our study was to describe and evaluate a novel technique which was originally developed in our department. Second, the inclusion criteria for this study were, however, focusing on bicondylar tibial fracture (AO/OTA type C). Actually, with the aid of bidirectional rapid reductor, this percutaneous technique could be applied for all types of tibial plateau fractures (Schatzker type I to VI). Another limitation was that the duration of follow-up in our protocol was short with the mean time of 16.5 months. We were not able to provide data concerning mid-term or long-term complication such as the traumatic arthritis related to this technique.

## Conclusion

Our observations suggest that the bidirectional rapid reductor facilitates the minimally invasive treatment for bicondylar tibial plateau fractures. This novel technique required limited soft-tissue exposure which was typically suitable for high-energy related fractures. The patients exhibited excellent functional recovery. However further comparatives studies with a higher number of patients and longer follow-up are needed to confirm our short-term data.

## Abbreviations

ACL: Anterior cruciate ligament
LCP: Locking compression plate
MIPPO: Minimally invasive percutaneous plate osteosynthesis
ORIF: Open reduction and internal fixation
PCL: Posterior cruciate ligament
PSA: Posterior slope angle
TPA: Tibial plateau angle
